# Difficulties in choosing the right intraocular lens in a previously vitrectomized patient - the role of the tear film

**DOI:** 10.22336/rjo.2022.18

**Published:** 2022

**Authors:** Monica Mălăescu, Bogdana Tăbăcaru, Horia Tudor Stanca

**Affiliations:** * Ophthalmology Clinic, “Prof. Dr. Agrippa Ionescu” Clinical Emergency Hospital, Bucharest, Romania; ** Department of Ophthalmology, Faculty of Medicine, “Lucian Blaga” University, Sibiu, Romania; *** Department of Ophthalmology, “Carol Davila” University of Medicine and Pharmacy, Bucharest, Romania

**Keywords:** cataract, cornea, corneal ectasia, cylinder, keratoconus, monofocal IOL, tear film

## Abstract

We present the difficulties in choosing the right IOL, when facing a great variability of the keratometric measurements, in the case of a patient operated for epiretinal membrane and lamellar macular hole, who developed complicated cataract in the operated eye. Upon commencing the biometric measurements, inconsistency in keratometric values led to further investigations. Repeated placido disc topography initially showed corneal ectasia, which posed a problem on selecting the right type of intra-ocular lens. Ocular surface pathology was suspected, and after treatment, the topography was repeated with a Scheimpflug topographer, that showed an improved keratometric profile. The surgical solution was to implant an aspheric monofocal IOL, in the bag, with extended depth of focus that enhances intermediate vision, disregarding the previous keratometric measurements. Refractive and functional outcomes were good.

In cases of biometric measurements that show inconsistency in keratometric values, ocular surface disease as well as corneal ectasia should be taken into consideration. The right implant should not be chosen based on a single measurement, but rather several measurements should be made and compared and the choice should not be made before treating the ocular surface.

## Introduction

Cataract surgery has become a procedure that usually has good postoperative results thanks to the current medical advancements. With the development of technology, target refractive results can be obtained more easily and patient expectations have risen together with the rise of biometric accuracy and improved postoperative results [**1**-**4**]. The choice of the proper intraocular lens after obtaining correct biometric measurements has therefore become of great importance [**5**]. However, sometimes, repeated measurements can vary. Basic steps such as performing a correct biometry are often disregarded due to the lack of time and patience. Regarding keratometric measurements, it is recommended to take at least three readings, to repeat if the difference in total keratometric power between eyes exceeds 1.5 diopters, and to avoid using the obtained data if there is ocular surface or corneal pathology [**6**].

## Case report

**Fig. 1:**
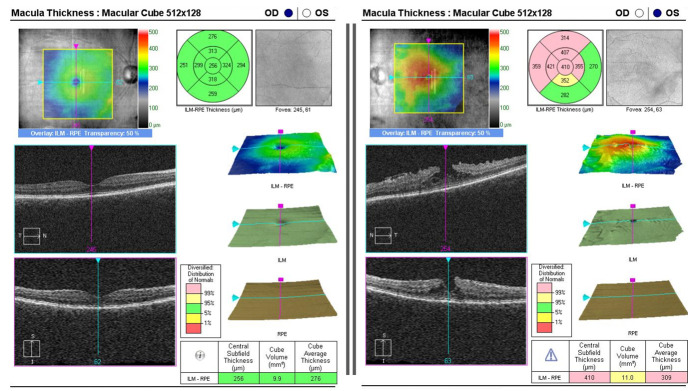
Optical coherence tomography (OCT) of the macula: right eye (OD) - normal macular contour; left eye (OS) - epiretinal membrane with traction on the foveal contour and lamellar macular hole

**Fig. 2:**
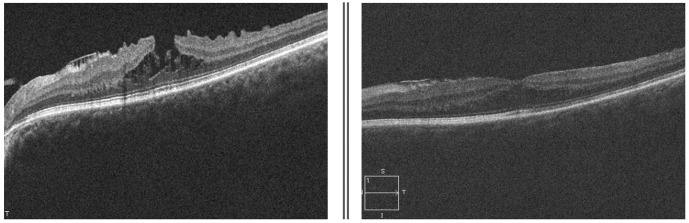
Epiretinal membrane with traction on the foveal contour and lamellar macular hole before (left image) and after (right image) surgery

A 68-year-old female patient was admitted in our clinic in 2019 for progressive decrease of visual acuity in the left eye. At presentation, her best corrected visual acuity was 20/ 20 (0 logMAR) for the right eye and 20/ 32 (0.2 logMAR) for the left eye with the appropriate correction (OD: +03.00 SD <> -00.50 CD 50; OS: +02.50 SD <> -01.75 CD 1700, where SD = spherical diopter, CD = cylindrical diopter). The intraocular pressure by Goldmann applanation tonometry (GAT) was 17 mmHg in the right eye and 12 mmHg in the left eye. The external examination and the anterior segment examination showed no abnormal findings, except for mild lens transparency changes in both eyes, with no influence on visual acuity. The fundus of each eye was examined after pharmaceutical mydriasis with 0.5% tropicamide and 10% phenylephrine hydrochloride ophthalmic solutions. The posterior segment examination revealed abnormal reflectivity of the macular area and fine wrinkling of the surface in the left eye. Optical coherence tomography (OCT) of the macula showed the presence of an epiretinal membrane with traction on the foveal contour and lamellar macular hole in the left eye (**Fig. 1**). The patient underwent posterior vitrectomy, epiretinal membrane peeling and internal gas tamponade with SF6 in the left eye with good anatomical and functional postoperative results, with 20/ 25 (0.1 logMAR) vision without correction (**Fig. 2**).

**Fig. 3:**
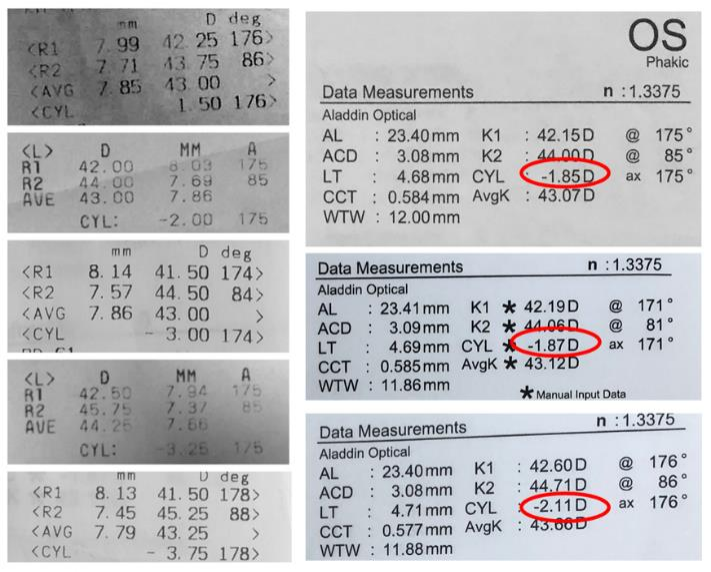
Left column - The keratometric cylinder measured on the auto ref/ kerato/ tono/ pachymeter Tonoref III (Nidek Co., Ltd.); Right column - Optical coherence biometry measured on the Aladdin HW3.0 biometer (Topcon)

**Fig. 4:**
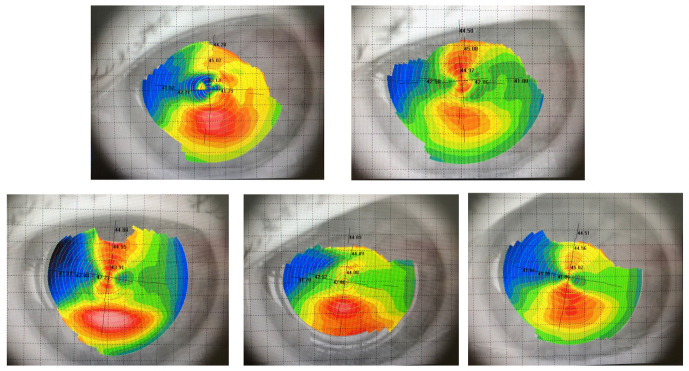
Variability of placido disc topography maps

**Fig. 5:**
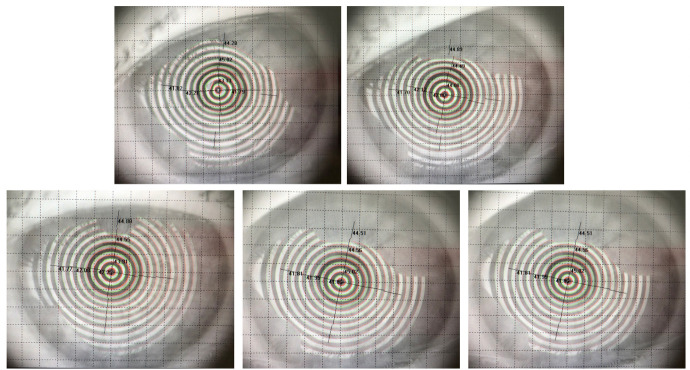
Irregularities of the rings projected on to the cornea during placido disc topography

**Fig. 6:**
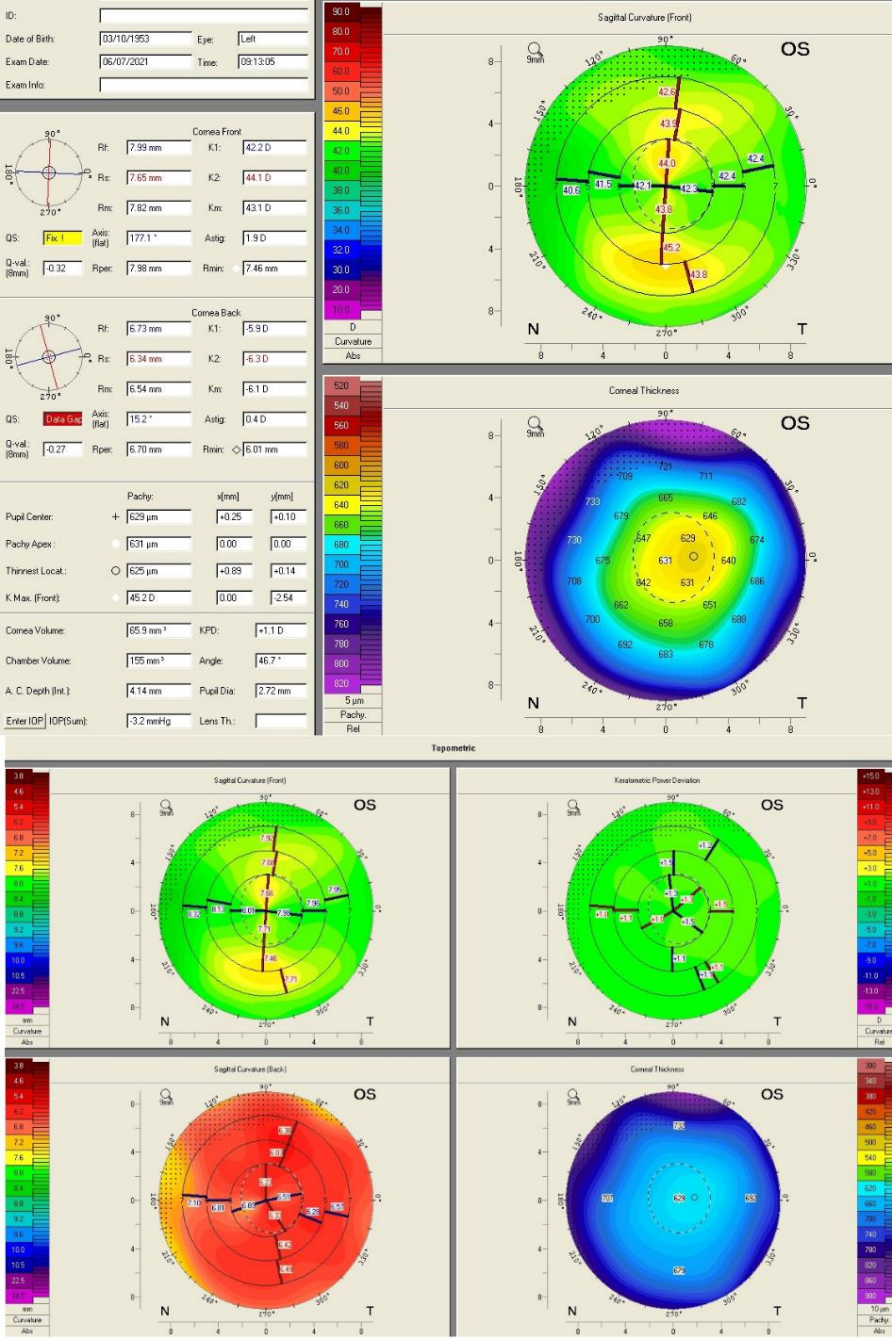
Scheimpflug topography

Three months after vitreoretinal surgery, the patient developed cataract in the operated eye. Upon commencing the biometric measurements, inconsistency in keratometric values were observed. The keratometric cylinder measured on the auto ref/ kerato/ tono/ pachymeter Tonoref III (Nidek Co., Ltd., Japan) varied from -01.50 cylindric diopters to -03.75 cylindric diopters (**Fig. 3**). The optical coherence biometry was assessed with Aladdin HW3.0 (Topcon, Japan). This also showed variability of the keratometry (**Fig. 3**), which led to further investigations. The built-in placido disc topography system of the Aladdin biometer showed a topography suggesting corneal ectasia in the inferior half of the cornea on some measurements (**Fig. 4**). However, the rings projected on the cornea were irregular in an unorganized matter, which led to the suspicion of ocular surface disease (**Fig. 5**). The patient received topical treatment for one month with hyaluronic acid 0.2% three times a day and retinyl palmitate 128 µg/ g once a day. Topographic measurements were repeated after treatment with a Scheimpflug topographer (Pentacam HR, Oculus, Inc., U.S.A), that showed a difference in keratometric values (**Fig. 6**). The cornea of the left eye presented with a with the rule astigmatism, with relative asymmetry of the bowtie in the lower half, but less conspicuous than the previous measurements. Considering the fluctuation of the keratometric values, an appropriate toric IOL could not be determined, and it was decided to implant a 22.5 diopter aspheric monofocal IOL with extended depth of focus that enhances intermediate vision (TECNIS® EyhanceTM). 

**Fig. 7:**
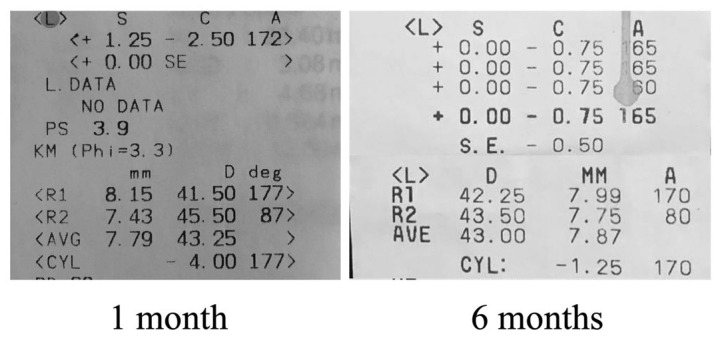
Refractometric and keratometric values at 1 month and 6 months after surgery

**Fig. 8:**
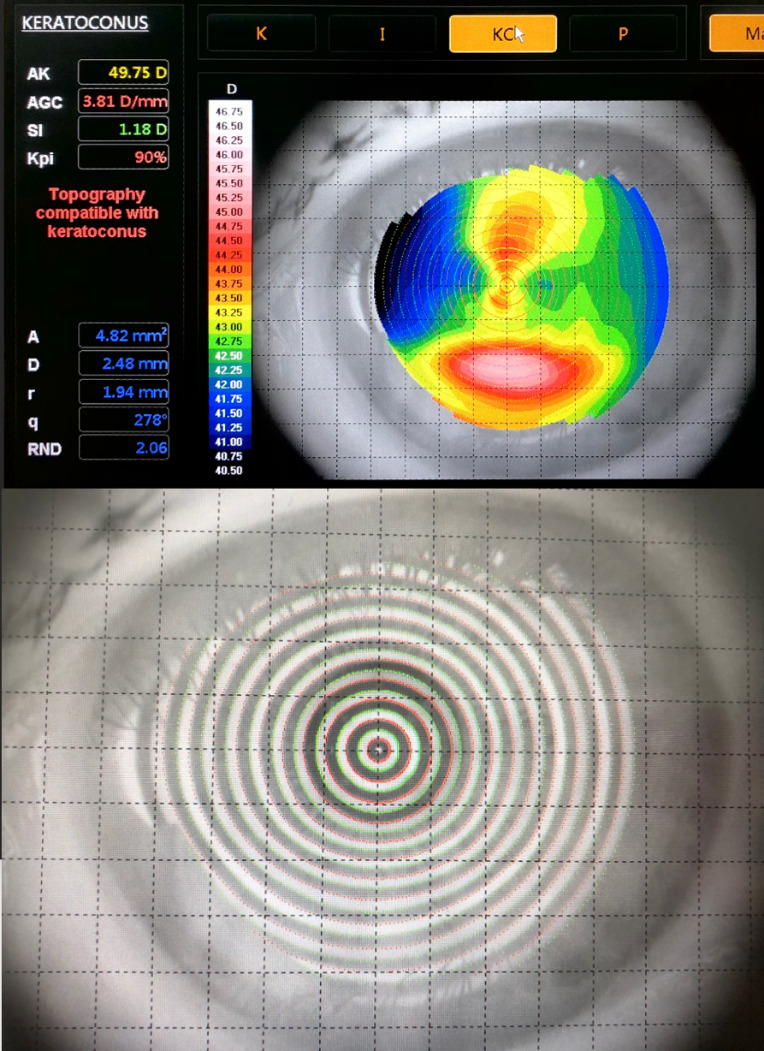
Placido disc topography 10 months after cataract surgery

At one month after surgery, the refraction of the left eye was +01.25 spheric diopters with -02.50 cylindric diopters at 1720 and a keratometric cylinder of -04.00 diopters. The spheric equivalent was 0 diopters. At 6 months, however, the refraction changed to 0 spheric diopters with -00.75 cylindric diopters at 1650, keratometric cylinder of -01.25 diopters and spheric equivalent of -00.50 diopters (**Fig. 7**). Refractive and functional outcomes were good, visual acuity being 20/ 25 (0.1 logMAR) without correction. All this time, we observed refraction instability, which was probably due to ocular surface irregularities. However, 10 months after the surgery, the placido disc topography showed a keratoconus prediction index of 90%, with no ocular surface irregularities that could influence the measurement (**Fig. 8**). The patient’s evolution will be followed up further, in order to assess the risk of progression.

## Discussion

The keratometry performed with the Aladdin biometer, which scans with placido disc technology was correlated with the keratometry obtained by the Tonoref TMIII, a device that measures via the double mire ring method. Additional keratometric measurement were performed with the Scheimpflug topographer Pentacam® HR.

Main disadvantages of placido-based topographers include the absence of information about the posterior corneal surface and limited corneal surface coverage omitting important data from the para-central and/ or peripheral corneal surface [**7**].

The Pentacam® HR takes up to 50 slit-images of the anterior segment of the eye in less than 2 seconds with a single Scheimpflug camera (rotating from zero to 180°). With these images, a three-dimensional image of the anterior surface is constructed [**8**]. The best advantage of Scheimpflug based devices over placido-based keratoscopes is the measurement of both the anterior and posterior corneal surfaces and global pachymetry in noncontact manner and it is a valuable tool in clinical practice, one of its most popular uses being the presurgical assessment [**7**].

Keratometric measurements can be influenced by irregularities in the tear film. The most powerful refraction occurs at the air-tear film interface where the greatest change in refractive index is from 1.00 to 1.34 [**9**]. It was estimated that changes to the anterior radius of curvature of the tear film in dry eye syndrome can result in up to 1.3 diopters power changes, that will give rise to refractive surprises. Through a simple calculation, Montés-Micó [**10**] illustrated the optical importance of the tear film in the imagery of an individual eye. The anterior radius of the tear film is approximately 7.8 µm, and its refractive index is 1.336, giving a surface power of 43.08 diopters (D). With a tear-film thickness between 6 µm and 20 µm, uniform reduction in the thickness of the film can have little effect on the surface power or aberrations because the surface radius can change by a maximum of only 20 µm to yield a maximum power increase of approximately 0.10 D. If, however, the film becomes irregular in thickness, much larger variations can occur in its local anterior radius of curvature and power [**10**]. Tear film instability can lead to significant variability of repeated keratometric measurements, which leads to variations in intraocular lens power calculations [**5**,**11**,**12**].

Tear film can be assessed before cataract surgery even without special measurements by analyzing the topography obtained during the biometric measurements of cataract patients. The mire rings projected onto the cornea during placido disc topography can show irregularities and patches of ring discontinuity if the tear film is not stable. The observation of these changes does not take much time for the evaluator and should be performed for each patient in order to ensure correct biometric measurements.

All biometric formulas require the keratometry and axial length as minimum parameters to evaluate necessary intraocular lens refractive power. Reproductible biometric measurements are hard to obtain in patients with corneal ectasia. The ratio between the anterior and posterior corneal curvature changes [**13**,**14**], the central corneal apex may be decentered. The axial length may be influenced by this, and the measured optical parameters of the eye can vary significantly [**14**-**17**]. Different corneal topography devices have different repeatability of the keratometric values that decrease in eyes with keratoconus [**18**-**21**]. This was proven for placido disc topography [**18**], as well as Scheimpflug devices [**19**]. Watson et al. showed that, in case of eyes with mean K under 55 D, the use of actual biometry derived keratometric values aiming for low myopia refractive target can offer acceptable refractive results [**22**]. Concerning this patient, mean keratometric measurements were acceptable and a good refractive result was expected. Toric intraocular lens implantation was not recommended in this case, even if studies have proven good visual acuity gain in patients with keratoconus [**23**,**24**], because the associated ocular surface pathology made the keratometric measurements even more unstable. Nevertheless, the refractive and visual acuity postoperative results were very good, with 20/ 25 (0.1 logMAR) vision without correction.

## Conclusion

In cases of biometric measurements that show inconsistency in keratometric values, ocular surface disease or corneal ectasia should be taken into consideration. The right implant should not be chosen based on a single measurement, but rather several measurements should be made and compared and the choice should not be made before treating the ocular surface if ocular surface disease is involved. 

**Conflict of Interest**

The authors state no conflict of interest. 

**Informed Consent and Human and Animal Rights statement**

Informed consent has been obtained from all individuals included in this study.

**Authorization for the use of human subjects**

Ethical approval: The research related to human use complies with all the relevant national regulations, institutional policies, is in accordance with the tenets of the Helsinki Declaration, and has been approved by the review board of “Carol Davila” University of Medicine and Pharmacy, Bucharest, Romania.

**Acknowledgements**

None.

**Sources of Funding**

None.

**Disclosures**

None.
